# Patients with chikungunya meeting criteria for inflammatory rheumatic diseases: a systematic literature review and meta-analysis

**DOI:** 10.1186/s42358-025-00471-6

**Published:** 2025-09-24

**Authors:** Viviane Angelina de Souza, Deivson Mendes Macedo, Nathalia Sernizon Guimarães, Mariana Peixoto Guimarães Ubirajara e Silva de Souza, Adriana Maria Kakehasi

**Affiliations:** 1 Universidade Federal de Juiz de Fora, Rua Flores de Ouro Preto, 221. Estrela Sul, Juiz de Fora, Minas Gerais, 36030-718 Brasil; 2 Universidade Federal de Minas Gerais, Belo Horizonte, Minas Gerais Brasil

**Keywords:** Arbovirus, Chikungunya, Meta-analysis, Rheumatic diseases, Systematic review

## Abstract

**Background:**

Chikungunya fever (CF) is marked by acute, subacute, and chronic phases, with a significant proportion of patients experiencing persistent joint and neuropathic pain. These symptoms may mimic those of rheumatic diseases such as rheumatoid arthritis (RA) and spondyloarthritis (SpA). There is currently no consensus on whether the infection directly causes chronic joint disease or serves as an immunological trigger for the development of rheumatic conditions.

**Aim:**

This study aims to evaluate the overall proportion of CF patients who progress to chronic arthropathy, and to identify how many of these patients meet the classification criteria for RA and SpA (including ankylosing spondylitis (AS) and psoriatic arthritis (PsA)).

**Methods:**

A thorough search was conducted in electronic databases, including PubMed, Embase, LILACS, and the Cochrane Library. The primary endpoint was the occurrence of chronic arthropathy, defined as joint signs and symptoms lasting more than six weeks following the acute phase of CF. The secondary endpoint involved the proportion of patients meeting the classification criteria for RA, SpA, AS, and PsA. A random-effects meta-analysis model was utilized to combine studies and determine the pooled frequency of persistent joint symptoms. Subgroup analyses were performed based on the fulfillment of classification criteria for rheumatic diseases. The risk of bias was assessed using the Joanna Briggs Institute critical appraisal tools. The study protocol was registered with PROSPERO (CRD42020211430).

**Results:**

A total of thirty-eight studies, comprising data from 12.524 individuals with CF published between 2008 and 2022, met the inclusion criteria. Of these, 4.324 (34.5%) patients developed chronic arthropathy; among them, 11.43% (240 in 2099) patients met the criteria for RA, 12.1% (86 in 711) patients for SpA, 3.42% (36 in 1052) patients for AS, and 2.05% (25 in 1220) patients for PsA.

**Conclusion:**

This study found that approximately one-third of CF patients experience persistent joint pain lasting over six weeks. However, only a minority of these individuals meet classification criteria for rheumatic diseases.

**Supplementary Information:**

The online version contains supplementary material available at 10.1186/s42358-025-00471-6.

## Background

Chikungunya virus (CHIKV) is a single-stranded RNA virus from the Togaviridae family and Alphavirus genus, transmitted primarily by Aedes aegypti and Aedes albopictus mosquitoes [1, 2]. Chikungunya fever (CF) poses a significant public health challenge in tropical and subtropical regions, where it causes debilitating joint and neuropathic pain [3]. The term “chikungunya” originates from the Makonde dialect, meaning “to bend forward,” reflecting the arthralgia and discomfort experienced by patients [4].

The global spread of CHIKV, from initial epidemics in Tanzania during the 1950s to recent outbreaks in the Americas, since 2014, highlights its impact [5]. A decade after the virus began circulating in the Americas, 3.7 million cases of CF have been reported across fifty countries and territories in the region, with Brazil accounting for 45% of these cases [6].

Progression to the chronic phase, which is characterized by symptoms persisting beyond six weeks, stems from both direct viral damage and inflammatory responses. These persistent symptoms resemble those of rheumatological diseases such as rheumatoid arthritis (RA), spondyloarthritis (SpA), and psoriatic arthritis (PsA) [7].

Pathophysiological changes post-CHIKV infection include synovial hyperplasia and macrophage infiltration, similar to those in autoimmune joint disorders [8]. Animal models further reveal bone tissue proliferation and necrosis [9]. Risk factors for chronicity include female gender, age over 45, and severe acute phase joint involvement [10, 11].

Chronic joint sequelae from CF not only mirror rheumatic diseases but also exacerbate morbidity and economic burdens, without specific vaccines or antivirals currently available [12]. This review evaluates the extent of chronic arthropathy in CF patients and assesses the overlap with established rheumatic disease criteria through observational studies.

## Methods

This systematic review and meta-analysis were adapted on recommendations from the Cochrane Guidelines for Systematic Reviews of Interventions taking into account the methodological guidelines set out by Borges Migliavaca et al., 2020 [13] and was written according to the Preferred Reporting Items for Systematic Reviews and Meta-Analyses (PRISMA) (Supplementary material 1) [14–16].

The review protocol was registered at the PROSPERO (CRD42020211430).

There was no need for approval from an ethics committee as this was a systematic review and meta-analysis study.

All authors contributed to this study. VAS, DMM, NSG, MPGUSS, and AMK designed the study, wrote, and revised the main manuscript. NSG conducted the technical aspects related to the systematic review and meta-analysis. VAS, DMM and AMK conducted the study selection and data extraction. NSG and DMM were responsible for the risk of bias and quality assessment.

### Search strategy

We searched four independent databases to conduct a comprehensive and specific bibliographic search for each database until October 2024: PubMed, Embase, Cochrane Central, and the Latin American and Caribbean Health Sciences Information (LILACS). Additionally, we manually searched for observational studies using the references of the selected studies as a sample. There was no language, date, document type, publication status, or geographic restriction for the inclusion of records. Descriptors were identified in Medical Subject Headings (MeSH), *Descritores em Ciências da Saúde* (Decs) and Embase Subject Headings (Emtree). The search strategy was adapted based on descriptors in each database and is presented in the Supplementary material 2.

### Outcomes

Primary endpoint included the occurrence of chronic arthropathy in patients with CF defined as articular signs and symptoms lasting more than six weeks after the acute infection, according to the Recommendations of the Brazilian Society of Rheumatology for diagnosis and treatment of CF [17]. The secondary outcome was the proportion of patients with chronic CF meeting classification criteria for RA, SpA, AS, or PsA, as following: ACR 1987 [18] and ACR/EULAR 2010 for RA [19], ASAS 2009, ACR and ESSG criteria for SpA [20], Modified New York criteria for AS [21] and CASPAR and ACR for PsA [22].

### Eligibility criteria

We included observational studies (cross-sectional, cohort, and case-control) that evaluated patients with confirmed CF (by clinical or laboratory testing) that presented chronic arthropathy and assessed any primary (more than six weeks of articular symptoms) and secondary outcomes (fulfillment of classification criteria for RA, AS, SpA or PsA). Randomized clinical trials, case series, case reports, systematic reviews with or without meta-analysis, narrative, and integrative reviews were excluded.

### Study selection and data extraction

Electronic search results from defined databases were uploaded to the Rayyan Qatar Computing Research Institute [23]. Study selection and data extraction were independently performed by two investigators. A third reviewer resolved any disagreements. For duplicate registrations, only the most recent one was included. Authors initially screened titles and abstracts. Subsequently, they assessed each study to determine whether it met inclusion criteria.

We extracted data on first author of the study, year of publication, study population (n, sex, age group, average, median value age), scenario in which the data were evaluated (outpatient/ hospital), study design, sample selection (sample calculation and sampling), number of events (chronic arthropathy, RA, SpA, AS, or PsA), classification criteria, outcomes and main results.

### Risk of bias and quality assessment

Two investigators (NSG and DMM) independently assessed the risk of bias in the selected studies according to the Joanna Briggs Institute (JBI’s) critical appraisal tools [24]. Three scores of yes, no, and unclear or Not/Applicable, referring to high risk, low risk, and unknown risk, respectively, were performed (Supplementary material 3). The reviewers resolved discrepancies by discussion.

### Sensitivity analysis

To assess the robustness of the pooled prevalence estimates, a sensitivity analysis was conducted. This involved the exclusion of studies with non-standard (laboratorial) diagnostic criteria, those classified as having higher risk of bias, and studies that reported 100% event rates, which could disproportionately influence the meta-analytic estimates. The sensitivity analysis aimed to evaluate the stability of the results under different analytical assumptions.

### Statistical analysis

A random effects model was used to calculate pooled frequency estimates of the number of patients with CF who presented with chronic arthropathy and those diagnosed with RA, SpA, AS or PsA. Pooled frequency estimates were expressed as mean estimates and 95% confidence intervals (CIs). Forest plots were used to visually assess the combined estimates and their corresponding 95% confidence intervals. We used an *I*^*2*^ statistic estimate of ≥ 50% as an indicator of large statistical heterogeneity.

We conducted the meta-analysis estimates that had been transformed using the raw proportions (PRAW) method with random effect. Subgroup analyses were performed by classification criteria. In all analyses, p-value < 0.05 was defined as statistically significant. Analyses were performed using the R language (4.3.2).

## Results

### Search results

**Fig. 1:**
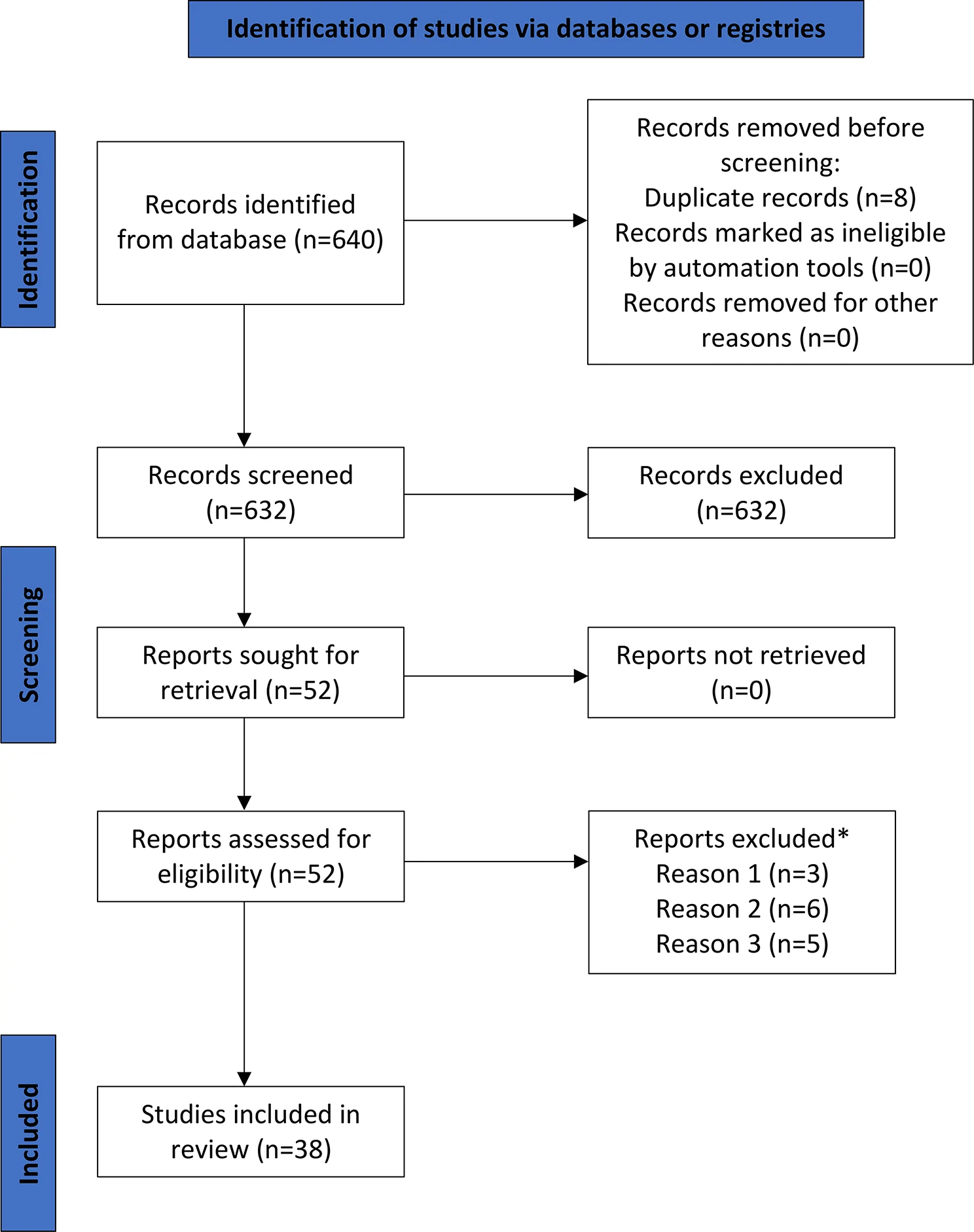
Flowchart of information through the different phases of the systematic review. The flowchart was adapted from the Preferred Reporting Items for Systematic Review and Meta-Analyses flowchart model. *Reason 1 - Wrong Research Design, i.e. the patient population or study design was inadequate; Reason 2 – Wrong Patient, i.e., did not include participants with chronic arthropathy; Reason 3 – Wrong Setting, i.e. did not meet the study design criteria

After the search, 640 references were identified. No study was found in LILACS. After excluding duplicate studies, 632 articles were included for screening of titles and abstracts. Of these, 580 were excluded because they did not meet the inclusion criteria (Supplementary material 4). Thus, 52 studies were included in the textual analysis. Six articles did not include participants with chronic arthropathy, 5 did not meet the study design criteria and in 3 the patient population was not suitable. According to the inclusion criteria, 38 studies were finally included in the qualitative and quantitative synthesis. Data that remained unrecoverable (when authors did not respond to contact) were not included in the meta-analysis. Figure 1 shows the results of the search and selection of studies.

Among the studies that reported gender distribution, the participants were predominantly female (68%). The papers selected for this systematic review were published between 2008 and 2022, and no studies were retrieved after that. In terms of geographical location, 12 studies were carried out in India, 6 in Colombia, 5 in the French Antilles, 4 in Brazil, 2 in France, 2 in the Philippines, 1 in Bangladesh, 1 in Jamaica, 1 in Pakistan, 1 in Puerto Rico, 1 in Singapore, 1 in Thailand, and 1 in Venezuela. As for the design of the studies included, 4 were case-control studies, 11 cross-sectional studies, and 23 cohort studies, comprising 38 studies. Of the 23 cohorts, 17 were prospective and 6 retrospective. Regarding the follow-up time of the cohorts, the lower limit was 7 weeks and the upper limit was 288 weeks, and the median follow-up time was 24 weeks. Most of the studies did not report ethnicity, in 1 Colombian study the majority were Afro-Colombian, in another the mestizos stood out and in 2 Brazilian studies black race predominated. Table 1 illustrates the results of the qualitative synthesis.

**Table 1:** Characteristics of the studies included in the qualitative synthesis

Reference, location	Study design	Sample (*n*)	Sex (female)	CF + Chronic Arthropathy	Mean age ± SD or median (range)	Objective	Results	Study duration (weeks)
Abella et al., 2019, Colombia	cross-sectional	94	72	94	57 ± 14.99	To identify the clinical and immunological characteristics, functional disabilities, and quality of life decline in a sample of Colombian patients with chronic arthropathy associated with chikungunya virus.	Significant functional and quality of life involvement of the affected patients, with almost one third presenting with arthritis in the first 9 months following the acute infection. Synovitis was identified in 29.8% of the cases at the time of the assessment, with a mean of 2.21 ± 1.26 swollen joints.	N.A.
Genaro et al., 2020, Brazil	cross-sectional	106	98	106	51.63 ± 12.52	To identify possibly fast, simple low-cost biomarkers to monitor chronic CF-induced articular disease. A case was defined as arthritis in 4 joints for more than 6 weeks associated with laboratorial confirmation of recent acute CF infection.	Negative association between the disease duration at the first medical evaluation after initial infection and the persistence of joint symptoms in these individuals. The presence of risk factors for chronic disease was detected in our samples; however, there was no evidence of correlation with the intensity of musculoskeletal manifestations.	N.A.
Banerjee et al., 2018, India	case-control	65	37	25	55 (35–76)	To evaluate the status of several standard oxidative stress markers and their correlation in chikungunya patients suffering with polyarthralgia. Persisting polyarthralgia was determined by clinical signs and symptoms presented from 10 to 30 days of infection.	Patients suffering from persisting polyarthralgia post-CF infection generates higher amount of intracellular ROS along with altered oxidative damage parameters and Siglec-9 expression on monocyte.	139
Bertolotti et al., 2020, French West Indies	prospective cohort	193	N.I.	87	N.I.	To assess the prevalence of chronic CF arthritis (CCA) at 48 weeks and to search for acute phase factors significantly associated with chronicity.	The overall prevalence of CCA was 52.1%. The reduced frequency of intravenous rehydration was associated with the development of CCA in univariate analysis. Thus, initial dehydration is one of the potential factors leading to the development of CCA. Enthesitis and tenosynovitis are risk factors significantly associated with CCA in univariate analysis.	48
Blettery et al., 2016, Martinica	prospective cohort	128	N.I.	47	64.1 (34–88)	To describe chronic CF manifestations seen during the outbreak in the Caribbean from December 2013 to January 2015.	21.1% of the patients progressed to a chronic form of seronegative RA; Thirteen/45 of the patients met criteria for PsA; 7/45 met modified New York criteria for AS; 6/45 had nonradiographic SpA; 19/45 had nonclassified forms of SpA, meeting the European Spondyloarthropathy Study Group criteria; 9 patients (7.0%) fibromyalgia according to American College of Rheumatology criteria.	52
Bouquillard et al., 2018, France	retrospective cohort	307	255	243	54 ± 12.6	To analyse the characteristics and progression of rheumatic manifestations in patients with post-CF joint pain.	Joint manifestations (arthralgia and arthritis) occurred preferentially in the extremities. Inflammatory involvement of the ankle seems to be particularly suggestive of CF infection, given its frequency and intensity. In some cases, the clinical presentation suggested the onset of RA. 13 (4%) patients, of the 328 patients initially examined, and 6.2% of the patients who presented with clinical signs of synovitis were diagnosed with RA (ACR 1987 criteria).	139
Chang et al., 2018, Colombia	cross-sectional	33	27	25	56 ± 10.0	To determine if chikungunya virus persists in synovial fluid after infection, potentially acting as a causative mechanism of persistent arthritis.	Initial symptoms of CF included joint pain (97%), swelling (97%), stiffness (91%), and fever (91%). The most commonly affected joints were the knees (87%), elbows (76%), wrists (75%), ankles (56%), fingers (56%), and toes (56%). Synovial fluid samples from all patients with chikungunya arthritis were negative for chikungunya virus on qRT-PCR, showed no viral proteins on mass spectrometry, and cultures were negative. Case and control plasma cytokine and chemokine concentrations did not differ significantly.	N.A.
Chang et al., 2018, Colombia	prospective cohort	485	388	123	49 ± 16	To estimate the frequency of chronic joint pain after CF in a Latin American cohort.	After 20 months, one-fourth of the participants had persistent joint pain. The most commonly affected joints were the small joints, including the wrists, ankles, and fingers.A multivariable analysis indicated that significant predictors of persistent joint pain included college graduate status, initial symptoms of headache or knee pain, missed work, normal activities affected, ≥ 4 days of initial symptoms, and ≥ 4 weeks of initial joint pain.	87
Chopra et al., 2012, India	prospective cohort	509	N.I.	202	N.I.	To evaluate long-term pain and chronic musculoskeletal rheumatic pain after CF.	Fifty-nine cases (12%;CI: 8.9–14.7) and 24 cases (5%, CI: 3.0–6.9) suffered from musculoskeletal pains and arthritis at 1 and 2 years, respectively. Thirteen cases tested seropositive only for rheumatoid factor: 11 non-specific arthralgias), one known RA and one OA.	104
Chopra et al., 2008, India	prospective cohort	156	105	95	N.I.	To evaluate the characteristics of musculoskeletal disorders of patients with CF.	Seventy-two patients (73%) had inflammatory arthritis. No classic erosions of RA on radiographs of the hands and feet of patients with inflammatory arthritis.	21
Chow et al., 2010, Singapore	case-control	30	26	4	39	To investigate the profiles of clinical disease, viral load, and immune mediators in patients with CF.	Arthralgia or joint pains persisted at the chronic phase (month 2–3 after illness onset) in 4 patients (13.3%). Persistent arthralgia was associated with higher levels of IL-6 and granulocyte macrophage colony stimulating factor.	7
Ganu et al., 2009, India	prospective cohort	16	9	16	50	To study clinical features and laboratory findings in patients with CF chronic arthritis.	Most commonly affected joints were wrists, MCPs, PIPs, elbows, shoulders, knees, ankles and MTPs. Out of 16 patients 2 were positive for RF (12.5%) and 9 were positive for anti-CCP (56.5%). X-Rays of hands and other affected joints revealed reduced joint space and periarticular osteopenia. Erosions developed in 5/16 patients (31.25%).	104
Gutierrez-Rubio et al., 2014, Philippines	prospective cohort	70	53	37	39.2 ± 13.50	To describe the course and outcome of musculoskeletal manifestations in patients with CF seen over a three-month period.	The most common joint areas involved were the ankles (60.0%), the wrists (40.0%) and the small joints of the hand (51.4%). Twenty-seven (47.3%) had symmetric arthritis. Thirty-seven cases (52.9%) had arthralgia or arthritis for at least six weeks. By the end of the follow-up period, only four (5.7%) had persistent musculoskeletal symptoms. Age and sex were not found to be factors in determining chronicity of arthritic symptoms.	12
Hayd et al., 2020, Brazil	cross-sectional	120	98	40	49	To describe the duration, severity, an characteristics of CF arthritis in comparison with local controls to further understand the long-term rheumatologic impact.	Four patients in the CF arthritis group were diagnosed with RA using the ACR criteria and did not have a pre-existing diagnosis of RA nor arthritis symptoms.	N.A.
Hoarau et al., 2010, France	prospective cohort	49	30	9	60 (19–90)	To identify prognostic markers of arthritis-like pathology after CF disease.	A polarized inflammatory immune response is engaged in the acute phase of CF although only weak circulating levels of proinflammatory Th1 effectors and immunoregulatory Th2 molecules were detected. This response persisted in some patients who went on to develop chronic arthralgia.	52
Javelle et al., 2015, France	retrospective cohort	159	121	112	51	To retrospectively document the clinical and therapeutic courses of rheumatic and musculoskeletal CF over a 6-year period.	Ninety-four patients (59%) who were free of any articular disorder prior to CF met the chronic arthropathy criteria: RA (*n* = 40), SpA (*n* = 33), undifferentiated polyarthritis (*n* = 21).	288
Manimunda et al., 2010, India	prospective cohort	203	107	100	35 (25–44)	To describe the acute (first month) and chronic (10th month) clinical features of CF disease, as well the nature of chronic arthritis.	Thirty four (36%) patients in the cohort met the ACR criteria for RA.	43
Mathew et al., 2011, India	prospective cohort	5277	313	437	48.37 ± 13.62	To describe the clinical profile of chronic rheumatic musculoskeletal pain and disorders, captured inadvertently about 15 months following a CF epidemic.	The most frequent diagnosis was chronic postviral polyarthralgia (57%). RA and seronegative SpA were observed in 6 and 11 patients, respectively. PsA was the commonest SpA, comprising 2.5% of the diagnoses.	60
Nayak et al., 2020, India	prospective cohort	184	6	30	46.1 (20–70)	To assess the antibody response patterns in CF acute febrile patients from India and then evaluated the impact of these febrile antibody response patterns on the downstream outcome of protection versus progression to chronic arthritis.	Thirty (100%) presented with arthralgias and 27 (90%) polyarthritis.	86
O’ Sullivan et al., 2018, Jamaica	cross-sectional	306	153	215	47.6 ± 18.5	To report demographic and self-reported clinical characteristics associated with persistent and severe arthralgia 8–12 months post CF infection.	70.3% (215) of patients reported arthralgia. Females and previous arthritis showed a greater likelihood for severe arthralgia as well as persistent arthralgia.	48.
Paul et al., 2011, India	prospective cohort	150	90	135	N.I.	To study the clinical profile of acute CF, chronic sequelae and response of these patients to treatment.	Rheumatoid factor was done in 44 (44%) patients with symptoms lasting more than 6 months and 15% (15 patients) had elevated values. Five patients turned out to have RA clinically and with anti-CCP positivity. Synovial biopsy was done for three patients with persistent arthritis and all showed features of chronic synovitis.	78
Rodriguez-Morales et al. [30], Colombia	retrospective cohort	283	173	283	29 (17–42)	To study the proportion of patients evolving to chronic polyarthralgia post CF.	Of the CF infected subjects, 152 (53.7%) reported persistent rheumatological symptoms. All of these patients reported joint pains (chronic polyarthralgia, 49.5% morning stiffness, 40.6% joint edema, and 16.6% joint redness.	26
Segura-Charry et al., 2020, Colombia	retrospective cohort	410	363	280	57.3 ± 11.7	To evaluate symptomatic and serological behaviour in patients suffering from CF infection, and to describe the comorbidities associated with the chronic phase of the disease.	46.3% of the cases presented persistent non-inflammatory arthralgias, and 53.7% of the patients underwent arthritis on physical examination. 20.3% (87 patients) were diagnosed de novo post-CF RA (58.6% of whom were seropositive and 11 patients (13.4%) were doubly seropositive for the RA test/antiCCP positive, and remarkably 14 patients (17%) were anti-CCP positive with a negative RA test); 7.3% (31 patients) were diagnosed with axial and/or peripheral SpA (15 patients with peripheral involvement, 11 patients with axial involvement and five patients with development of PsA), with the presence of HLA-B27 positive in 46.7%, 45.5% and 0% of cases, respectively.	195
Sissoko et al., 2009, France	retrospective cohort	147	102	84	52.0 ± 15.0	To assess the course of late rheumatic manifestations and investigating potential risk factors associated with the persistence of these rheumatic manifestations.	Age > 45 years, severe initial joint pain, and presence of underlying OA were predictors of nonrecovery.	15
Tritsch et al., 2019, Colombia	prospective cohort	485	N.I.	123	51.0 ± 14.0	To determine the frequency of chronic joint pain and stiffness 3 years after infection with CF.	54% of those patients reported continued joint pain 40 months after infection. Two patients with persistent joint pain developed RA after CF infection.	173
Alam et al., 2018, Pakistan	cross-sectional	52	37	52	45.0 ± 5.0	To study clinical and laboratory features associated with persistent arthralgia in patients with CF.	Mean duration of arthralgia was 2.6 months. Symmetrical arthralgia and asymmetrical was described in 76.9% and 23.1% of cases respectively. Small joint involvement was in 21.2%, large joint in 20.8% while both small and large involvement was seen in 48.1% of patients.	N.A.
Fuentes-Silva et al., 2017, Venezuela	prospective cohort	168	150	168	55.33 ± 12.72	To examine how many patients with CF evolve to chronic rheumatic diseases.	Thirty patients (17.85%) evolved into the following rheumatic diseases: RA (12.50%), PsA (2.38%), SpA (1.79%) and systemic lupus erythematosus (0.60%). There was a statistically significant association between positivity of RF, anti-CCP or both with development of the de novo rheumatic diseases.	104
Gutierrez-Rubio et al., 2014, Philippines	prospective cohort	70	53	37	39.2 ± 13.50	To describe the course and outcome of musculoskeletal manifestations in patients with CF seen over a three-month period.	The most common joint areas involved were the ankles (60.0%), the wrists (40.0%) and the small joints of the hand (51.4%). Twenty-seven (47.3%) had symmetric arthritis. Thirty-seven cases (52.9%) had arthralgia or arthritis for at least six weeks. By the end of the follow-up period, only four (5.7%) had persistent musculoskeletal symptoms.	12
Gupta et al., 2018, India	prospective cohort	882	484	568	53.64 ± 15.82	To study the rheumatological manifestations of CF infection.	Fourty (5.4%) patients developed new onset knee OA, 38 (5.14%) met ACR/EULAR 2010 criteria for RA, 28 (3.78%) met modified New York criteria for AS, 29 (3.92%) developed undifferentiated arthritis, 17 (2.3%) patients developed various enthesitis and tenosynovitis, 1 (0.14%) patients fulfilled ACR 1997/SLICC 2012 criteria for SLE, 20 (2.7%) patients fulfilled ACR 2010 criteria for fibromyalgia, 5 (0.67%) patients fulfilled CASPAR criteria for PsA, 3 (0.4%) patients fulfilled 2015 classification criteria for gout.	47
Alam et al., 2018, India	cross-sectional	112	81	37	N.I.	To study clinical and laboratory features associated with persistent arthralgia in patients with CF.	Symmetrical arthritis was reported in 85 (75.9%) patients, while asymmetrical arthritis was seen in 27 (24.1%) patients. 75 (66.9%) had polyarthralgia involving small, medium and large joints. Morning stiffness was reported in 68 (60.7%) of patients. Three patients later after 6 months of follow-up had anti-CCP positive, while 2 were negative. They were treated as inflammatory arthritis with DMARDS.	24
Patel et al., 2019, India	case-control	400	N.I.	24	N.I.	To evaluate the prevalence of post CF chronic arthritis among 400 consecutive acute coronary syndrome patients.	This study is an abstract and does not provide further details about the joint involvement of patients, only the occurrence of chronic arthropathy following CF infection.	N.I.
Rahim et al., 2010, India	retrospective cohort	342	N.I.	122	N.I.	To study the factors associated with persistence of pain post CF attack.	Major sites of persistent pain were noted to be knee, ankle, wrist and elbow with predisposition to the right side of the body.	32
Salgado et al., 2018, Brazil	cross-sectional	76	65	76	57.17 (standard deviation not informed)	To evaluate the prevalence of rheumatologic manifestations in 76 patients with CF infection, during an epidemic that occurred in 2016 in the city of Rio de Janeiro, Brazil.	Polyarthralgia occurred with higher prevalence (39 patients, 51,31%), affecting wrists, ankles, shoulders, knees and hands. Twenty six patients had arthritis (34,21%), Tendinopathy in 27 regions, being more common in shoulders, ankles and wrists and including De Quervain. Paresthesias occurred in 7 patients, prevailing carpal tunnel syndrome and one patient presented dactylitis.	N.A.
Benjamanukul et al., 2021, Thailand	cohort	164	121	91	48,2 ± 14.0	To describe the rheumatic manifestations in CF infection, estimate the prevalence of enthesitis in CF infected patients, and determine the factors associated with CF induced enthesitis.	48 patients (52.7%) had post-CF chronic inflammatory rheumatism, including 7 (14.6%), 11 (22.9%), and 30 (62.5%) who were classified as having RA, SpA, and undifferentiated arthritis, respectively. Forty-three patients (47.3%) had post-CF muskuloskeletal disorder. Of these, 14 (32.6%) displayed diffuse symptoms (polyarthralgia in 12 patients and polyenthesitis in 2). Twenty-nine (67.4%) patients had locoregional symptoms (monoarthralgia ou oligoarthralgia in 18 patients, neuropathic pain in 6, enthesitis in 4, and tendinitis in 1).	N.A.
Bossy et al., 2020, French Carribean	cross-sectional	61	51	61	N.I.	To describe the clinical profile in a cohort of patients in Guadeloupe with chronic clinical manifestations 36 months after CF infection.	Beside arthralgia, joint stiffness and general symptoms (asthenia, sleep disorders) contribute to the impairment of QoL lasting years after chikungunya.	144
Hossain et al., 2022, Bangladesh	prospective cohort	143	60	60	43,3 ± 11,5	To identify the clinical patterns and consequences of post-CF arthritis.	Among the 60 chronic CF patients, 35 had undifferentiated arthritis, where polyarthralgia was present in 21 (60.0%) patients, and polyarthritis was present in 8 (22.8%) patients. Ten patients had SpA where polyarthralgia was present in 6 (60.0%) patients, and seven patients had RA where 5 (71.4%) had polyarthritis.	52
Medina-Cíntron et al., 2021, Puerto Rico	case-control	61	41	33	52,5 ± 10,5	To evaluate the clinical features and correlates in DMARD-naive patients with chronic CF arthritis.	Joints of upper extremities were more commonly involved than those of lower extremities. Joint tenderness was most common in MCP joints (54.6%), shoulders (51.5%), PIP (45.5%), and knees (39.4%). Joint swelling was more commonly observed in PIP joints (15.2%), MCP joints (12.1%), shoulders (9.1%), wrists (6.1%), and ankles (6.1%). Nineteen (57.6%) patients with chronic CF arthritis had moderate to high disease activity.	24
Watson et al., 2021, Brazil	cross-sectional	40	36	40	49.5 (42–55)	To compare the relevance of joint counts and symptoms to clinical outcomes in RA and chronic CF disease.	Only stiffness was significantly associated with disability in CF patients. In CF arthritis, tender joint counts were significantly correlated only with one quality of life domain, while pain intensity score correlated with impaired mobility, self-care and pain/discomfort domains, and stiffness was associated with the mobility, usual activity and anxiety/depression domains.	108

N.A. = not applicable; N.I. = not informed; CF: chikungunya fever; pCHIK-CIR = post-CHIKV chronic inflammatory rheumatism; pCHIK-MSD = post-CHIKV musculoskeletal disorders; RA: rheumatoid arthritis; SpA: spondyloarthritis; PsA: psoriatic arthritis; OA: osteoarthritis; CCP: cyclic citrullinated peptide; MCP: metacarpophalangeal; PIP: proximal interphalangeal joints; MTP: metatarsophalangeal

*Spondyloarthritis (SpA). Undifferentiated polyarthritis (UP). Patients who had not previously displayed*,* rheumatic symptoms and developed CIR immediately after CF were classified as de novo pCF*,* CIR.Patients who had not previously displayed rheumatic symptoms and developed SA immediately after*,* CF were classified as de novo pCF-SpA*

σ^2^= 1108,64 / 22

σ^2^= 50,39

σ^2^= 7,09

### Meta-analysis

**Fig. 2:**
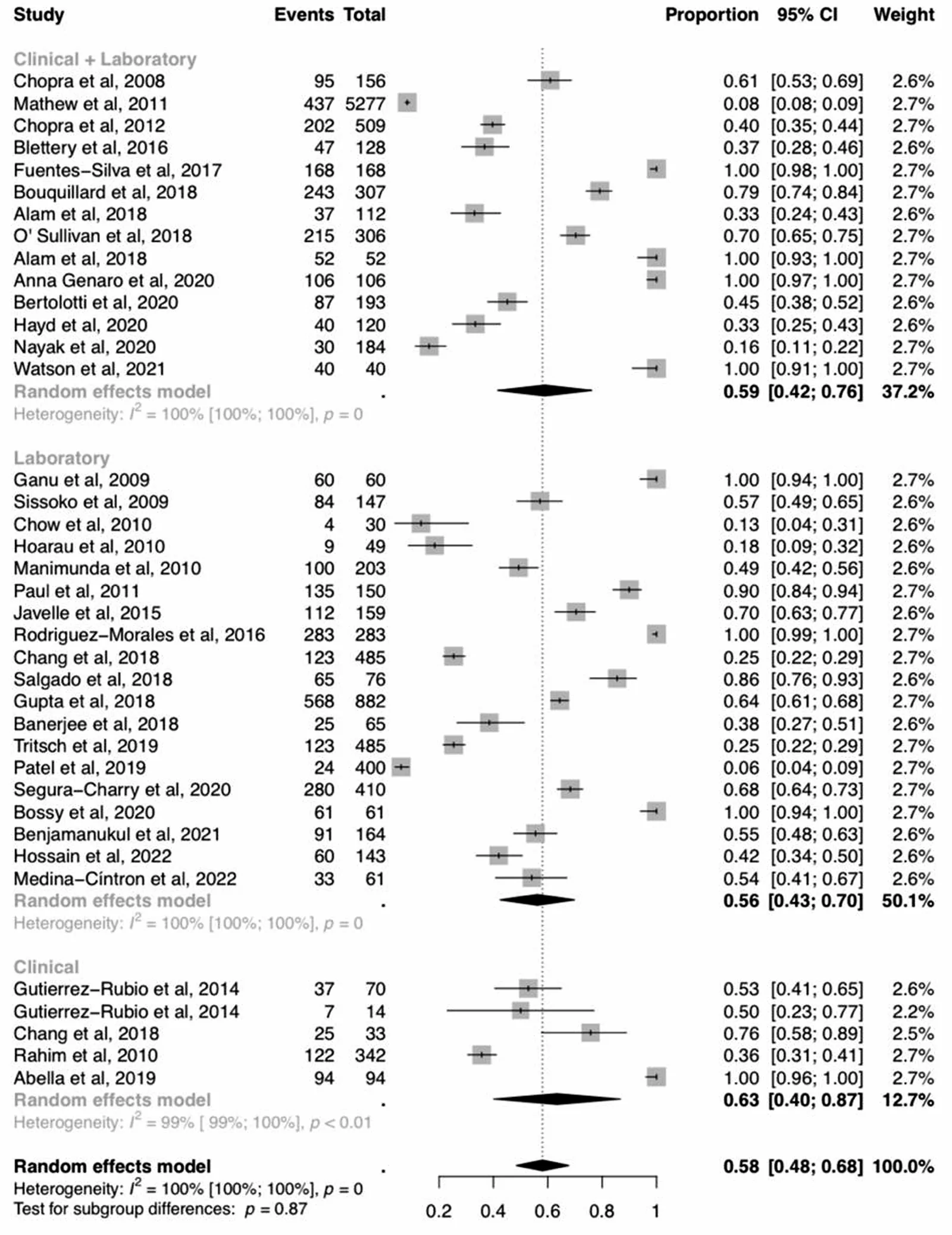
Proportion of patients with Chikungunya fever with chronic arthropathy and met clinical and/or laboratorial criteria, divided into subgroups

The diagnosis of CF in patients with chronic arthropathy was clinical-laboratorial in 59.0% (95%CI 42.0–76.0) of patients, laboratorial in 56.0% (95%CI 43.0–70.0) and clinical in 63.0% (95%CI 40.0–87.0). In total, 12,524 individuals with CF were included and, of these, 58.0% (95%CI 48.0–68.0) developed chronic arthropathy (Fig. 2).

The meta-analysis values are shown in Supplementary material 5.

### Sensitivity analysis

For this analysis, we excluded the studies of Rodriguez-Morales et al., 2016; Bossy et al., 2020, and Ganu et al., (2009). We found that the pooled prevalence estimates remained consistent under these analytical assumptions, suggesting that our findings are robust despite the high heterogeneity (Supplementary material 6).

**Fig. 3:**
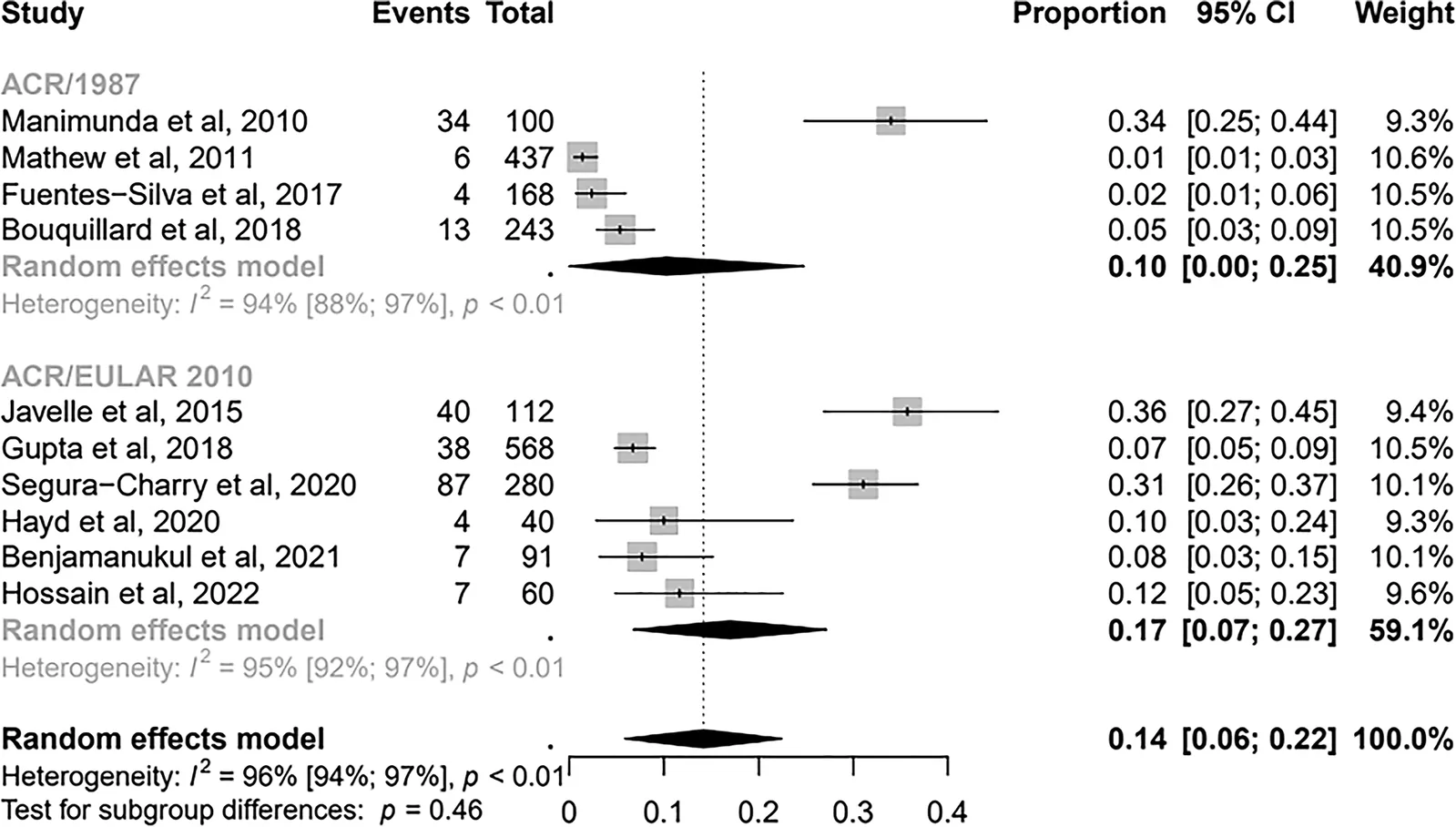
Frequency of patients with Chikungunya fever and chronic arthropathy who fulfilled classification criteria to rheumatoid arthritis

The frequency of patients with CF who developed chronic arthropathy and met classification criteria for RA was 14.0% (95%CI 6.0–22.0). Of these, 23.75% (*n* = 57) obtained the criteria through ACR/1987 and 76.25% (*n* = 183) by ACR/EULAR **(**Fig. 3**).**

**Fig. 4:**
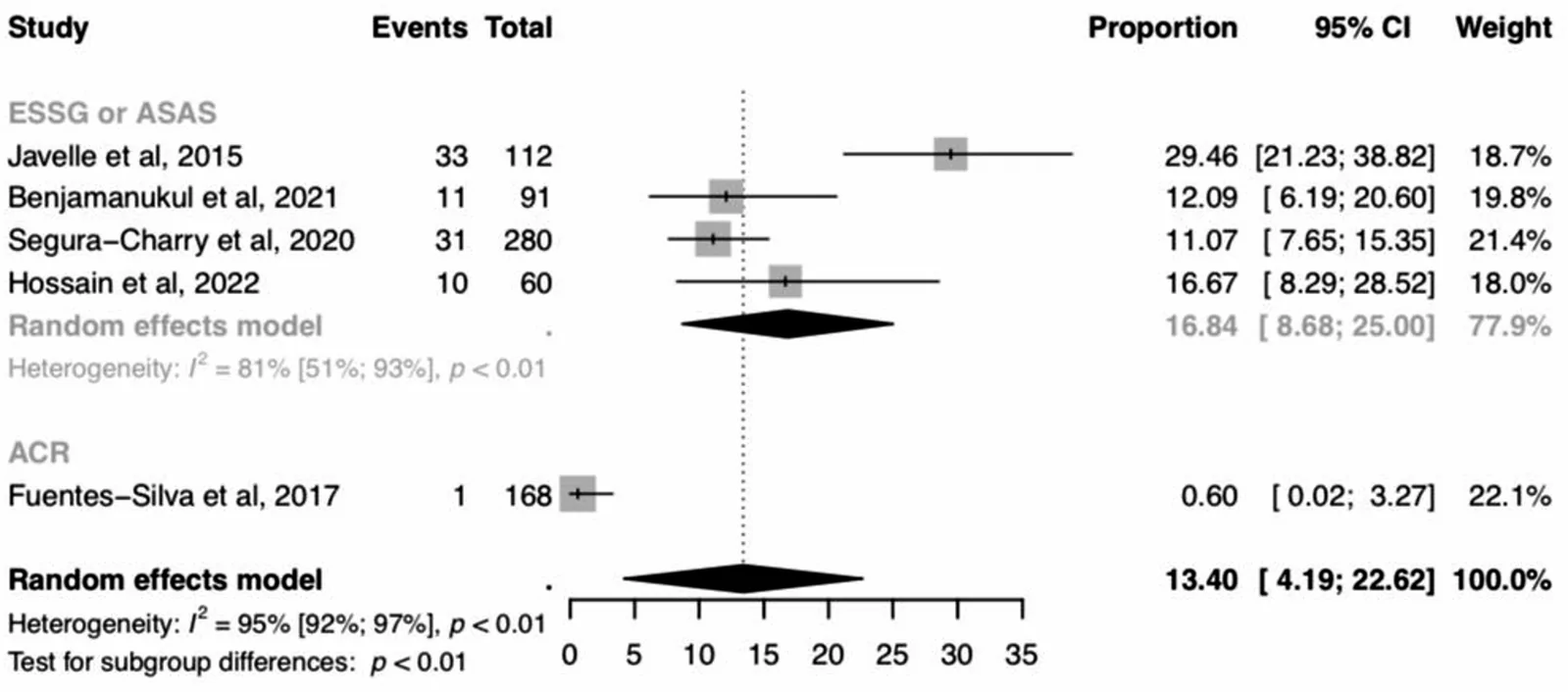
Frequency of patients with Chikungunya fever and chronic arthropathy who fulfilled classification criteria to spondyloarthritis

The frequency of patients with CF who developed chronic arthropathy and met criteria for SpA was 13.0% (95%CI 4.0–23.0). Of these, 51.2% (*n* = 44) were classified by ESSG criteria, 47.7% (*n* = 41) by ASAS criteria, and 1.16% (*n* = 1) by ACR **(**Fig. 4**).**

**Fig. 5:**
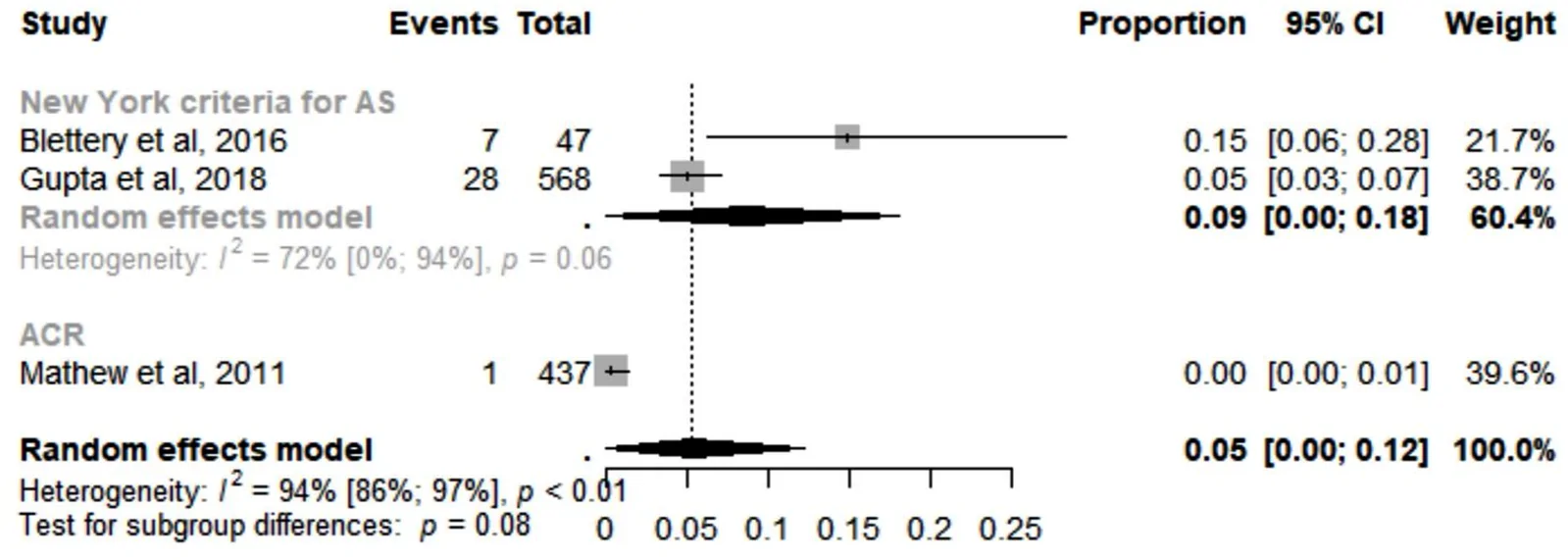
Frequency of patients with Chikungunya fever and chronic arthropathy who fulfilled classification criteria to ankylosing spondylitis

The frequency of patients with CF who developed chronic arthropathy and met criteria for AS was 5.0% (95%CI 0.0–12.0). Of these, 97.2% (*n* = 35) were classified by NY criteria and 2.8% (*n* = 1) for ACR (Fig. 5).

**Fig. 6:**
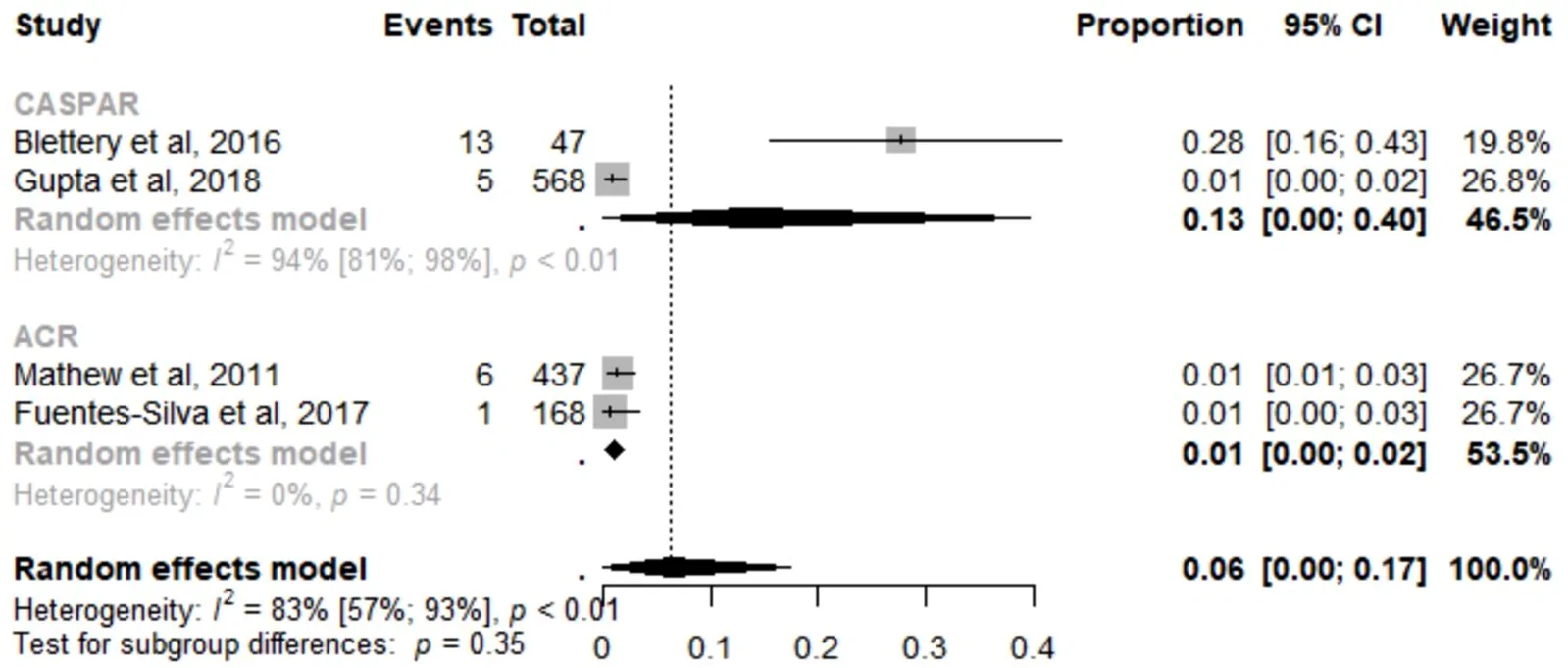
Frequency of patients with Chikungunya fever with chronic arthropathy and fulfilled classification criteria to psoriatic arthritis

The frequency of patients with CF who developed chronic arthropathy and met criteria for PsA was and 6.0% (95%CI 0.0–17.0), 72% (*n* = 18) classified by CASPAR criteria and 28% (*n* = 7) by ACR (Fig. 6).

## Discussion

This review synthesized evidence from observational studies on CF patients progressing to chronic arthropathy, evaluating classification criteria for RA, SpA, AS, or PsA. Our findings indicate a 58.0% frequency of chronic arthropathy in CF patients. Studies like Rodriguez-Morales et al. [25] report a 40.0% prevalence, while Edington et al. suggest 52.0% chronic joint symptoms post-infection, underscoring the need for targeted interventions [26].

The analysis by Paixão et al. of 38 articles revealed that 43.0% of patients with CF experienced arthralgia symptoms for more than three months [10]. This highlights the relevance of the condition in a significant subgroup of infected individuals. Complementing these findings, the systematic review of 37 studies conducted by Aalst et al., found that the persistence of arthralgia after CF ranged from 5.0 to 60.8%, depending on the follow-up period, with higher percentages observed in studies with closer monitoring over 13 months [27]. These variations in the frequency of chronic arthropathy between our results and those observed in the literature may be attributed to various factors, including selection bias among the studies, differences in host immune responses, viral mutations, and the genetic make up of the affected population [28]. This context emphasizes the need for further investigation to understand the determinants of symptom persistence across different patient cohorts.

In our study, among the patients who developed chronic arthropathy, the following percentages met the classification criteria: 14.0% for RA, 13.0% for SpA, 6.0% for PsA and 5.0% for AS. Nevertheless, chronic arthropathy following CF shares several characteristics with joint manifestations observed in immune-mediated rheumatic diseases. For instance, in RA, there is a notable prevalence among middle-aged women, alongside specific patterns of joint involvement and radiographic and laboratory findings that resemble those seen in chronic arthropathy post-CF. Additionally, the expression of pro-inflammatory cytokines and the response to various disease-modifying antirheumatic drugs (DMARDs) are comparable [29].

Several cohorts have reported patients with chronic arthropathy after CF that exhibit symptoms similar to those of RA or SpA [30–34]. A cross-sectional study conducted in Brazil identified that four out of 120 patients were diagnosed with RA following CHIKV infection [33]. Furthermore, Manimunda et al. found in a prospective cohort that 34 out of 94 patients met the criteria for RA [34].

It is noteworthy that almost a quarter of patients with a classification for RA met the 1987 ACR criteria, since the presence of bone erosion is one of the conditions required, and indicates a more established disease, with the possibility of irreversible joint damage. Unfortunately, we do not have data on the presence or frequency of radiographic changes in these studies.

Despite the clinical similarities, the radiographic involvement in CF arthropathy is still controversial. As mentioned, studies indicate that up to 40.0–60.0% of patients with CF may progress to the chronic form, but not all exhibit significant radiographic changes. Damage tends to be more evident in patients with risk factors such as advanced age, the presence of pre-existing joint comorbidities, or greater severity during the acute phase of the disease [8]. These data support the need for more studies to evaluate the radiographic progression of chronic CF arthropathy.

The mechanisms underlying the progression to the chronic phase of chikungunya infection are an area of ongoing investigation. It is recognized that the acute immune response to the infection plays a crucial role in the development of chronic arthropathy and the subsequent evolution into rheumatic diseases [35]. Studies have demonstrated that individuals experiencing chronic arthralgias exhibit aberrant immune responses, characterized by elevated levels of interleukin (IL)-6 compared to those who have recovered from the initial infection [29]. This suggests a persistent imbalance in the ratio of receptor activator of nuclear factor kappa-B ligand (RANKL) to osteoprotegerin (OPG), which may lead to ongoing bone erosions [36].

Additionally, the non-structural proteins (nsPs) produced by the CHIKV during host cell infection can neutralize various intrinsic antiviral responses. This results in limitations on the activation of antiviral genes, such as interferon beta (IFN-B), due to suppression of genomic transcription and interference with the JAK-STAT signaling pathway [37]. It is also noteworthy that in cases of chronic CF, approximately 95.0% of the reported pain mechanisms are non-inflammatory [38], which adds another layer to the complexity of the condition.

Unlike previous reviews, our study uniquely integrates data across continents, allowing for a comprehensive understanding of CF’s impact on rheumatic disease development. This broad approach offers insights into global epidemiological patterns and potential region-specific interventions.

We also selected studies explicitly addressing chronic arthropathy and rheumatic classification criteria, excluding those lacking robust diagnostic methodologies. This focus ensures clearer connections between CF and rheumatic conditions, contributing to a more targeted understanding that was absent in earlier reviews.

Our study presents limitations. We observed a high heterogeneity (> 90%) in the studies included in our analysis, which reflects the natural variation in prevalence across different contexts. Previous meta-analyses have also reported high heterogeneity (> 75%)in this field [25, 39]. We believe that excluding studies based solely on their prevalence estimates would potentially introduce selection bias and reduce the comprehensive nature of our review, which aims to capture the full spectrum of prevalence across different contexts. We conducted a sensitivity analysis excluding studies that did not classify CF according to laboratory criteria, those with a higher risk of bias, and studies that reported 100% event rates. Despite these exclusions, the pooled prevalence estimates remained consistent under these analytical assumptions, suggesting that our findings are robust despite the high heterogeneity. Variations in prevalence estimates across studies are generally expected due to genuine differences in populations, settings, and methodologies, rather than representing outliers or dispersed effects that would warrant exclusion [40, 41]. Future comprehensive cohort studies, accounting for regional disparities and detailed classification criteria, are essential to strengthen the evidence base and guide effective intervention strategies.

The risk of bias assessment for the cohort studies showed wide variability in methodological quality, with positive highlights for Bertolotti et al. (2020) and Hossain et al. (2022), who presented a low risk of bias across most domains, while the research conducted in India and Latin America demonstrated a higher proportion of domains with uncertain or high risk. Studies with higher risk of bias included those by Bouquillard et al. (2018, France), Chopra et al. (2008, India), Paul et al. (2011, India), and Fuentes-Silva et al. (2017, Venezuela), with moderate to high risk of bias. Rahim et al. (2010, India) also showed a high risk of bias. When evaluating the case-control studies, we noted that Medina-Cíntron et al. (2021, Puerto Rico) stood out for presenting a low risk of bias in all assessed domains, whereas Patel et al. (2019) and Chow et al. (2010) displayed higher methodological risk, reinforcing the heterogeneity in study quality. The cross-sectional studies presented various categories of risk of bias. Medina-Cíntron et al. (2021, Puerto Rico) and Watson et al. (2021, Brazil) showed low risk of bias, while Alam et al. (2018, Pakistan), Salgado et al. (2018, Brazil), and Bossy et al. (2020, French Caribbean) exhibited significant methodological limitations. This variability underscores the need for caution in interpreting findings and comparing studies from different geographical and methodological contexts (Supplementary material 3).

Another limitation is that some studies did not mention fulfillment of classification criteria, although some calculated composite disease activity indices. We recognize that classification criteria are tools for use in clinical trials, with the purpose of homogenizing patients. Nevertheless, it is significant to observe that some patients meet classification criteria, and this will be relevant in the discussion to evaluate the need for developing specific classification criteria for CHIKV arthropathy, and in the design of clinical trials that will evaluate disease-modifying medications for this group of patients.

## Conclusion

This review showed that a significant number of patients with CF experience persistent arthropathy. These symptoms may mimic those of rheumatological conditions such as RA and SpA. This review represents a pivotal step in understanding CF impact and its intersections with rheumatic disease progression. By highlighting these associations, we lay the groundwork for future research and clinical practices aimed at improving patient outcomes in affected regions.

## Supplementary Information

Below is the link to the electronic supplementary material.

Supplementary Material 1

Supplementary Material 2

Supplementary Material 3

Supplementary Material 4

Supplementary Material 5

Supplementary Material 6

## Data Availability

No datasets were generated or analysed during the current study.

## Declarations

### Data access, responsibility, and analysis

The lead authors DM and VAS had full access to all the data in the study and take responsibility for the integrity of the data and the accuracy of the data analysis.
