# Supplementary material for: Patients with chikungunya meeting criteria for inflammatory rheumatic diseases: a systematic literature review and meta-analysis
Source: Adv Rheumatol. 2025 Sep 24;65:38. doi: 10.1186/s42358-025-00471-6 (PMC13488800; doi:10.1186/s42358-025-00471-6)
Supplement: Supplementary file 2 [file 42358_2025_471_MOESM2_ESM.docx]

**Supplementary material 2 – Search strategy**

**Research Algorithms**

**PubMed/Medline**

*((Chikungunya vírus [Mesh] OR Chikungunya Fever [Mesh] OR Chikungunya Fevers OR Fever, Chikungunya OR Chikungunya Virus Infection OR Chikungunya Virus Infections OR Infection, Chikungunya Virus OR Chickungunya Fever OR Chickungunya Fevers OR Fever, Chickungunya) AND (Inflammation [Mesh] OR Inflammations OR Innate Inflammatory Response OR Inflammatory Response, Innate OR Innate Inflammatory Responses OR "inflammatory arthropathy")) AND (Arthritis, Rheumatoid [Mesh] OR Rheumatoid Arthritis OR Spondylitis, Ankylosing [Mesh] OR Spondyloarthritis Ankylopoietica OR Ankylosing Spondylarthritis OR Ankylosing Spondylarthritides OR Spondylarthritides, Ankylosing OR Spondylarthritis, Ankylosing OR Ankylosing Spondylitis OR Spondylarthritis Ankylopoietica OR Bechterew Disease OR Bechterew's Disease OR Bechterews Disease OR Marie-Struempell Disease OR Marie Struempell Disease OR Rheumatoid Spondylitis OR Spondylitis, Rheumatoid OR Spondylitis Ankylopoietica OR Ankylosing Spondyloarthritis OR Ankylosing Spondyloarthritides OR Spondyloarthritides, Ankylosing OR Spondyloarthritis, Ankylosing OR Spondylarthritis [Mesh] OR Spondylarthritides OR Spinal Arthritis OR Spinal Arthritides OR Arthritis, Spinal OR Arthritis, Psoriatic [Mesh] OR Psoriasis, Arthritic OR Arthritic Psoriasis OR Psoriatic Arthritis OR Psoriasis Arthropathica OR Psoriatic Arthropathy OR Arthropathies, Psoriatic OR Arthropathy, Psoriatic OR Psoriatic Arthropathies OR Arthritis [Mesh] OR Arthritides OR Polyarthritis OR Polyarthritides) AND (((((((((("chikungunya virus"[MeSH Terms] OR "chikungunya fever"[MeSH Terms]) OR (((("chikungunya fever"[MeSH Terms] OR ("chikungunya"[All Fields] AND "fever"[All Fields])) OR "chikungunya fever"[All Fields]) OR ("chikungunya"[All Fields] AND "fevers"[All Fields])) OR "chikungunya fevers"[All Fields])) OR (((("chikungunya fever"[MeSH Terms] OR ("chikungunya"[All Fields] AND "fever"[All Fields])) OR "chikungunya fever"[All Fields]) OR ("fever"[All Fields] AND "chikungunya"[All Fields])) OR "fever chikungunya"[All Fields])) OR (((("chikungunya fever"[MeSH Terms] OR ("chikungunya"[All Fields] AND "fever"[All Fields])) OR "chikungunya fever"[All Fields]) OR (("chikungunya"[All Fields] AND "virus"[All Fields]) AND "infection"[All Fields])) OR "chikungunya virus infection"[All Fields])) OR (((("chikungunya fever"[MeSH Terms] OR ("chikungunya"[All Fields] AND "fever"[All Fields])) OR "chikungunya fever"[All Fields]) OR (("chikungunya"[All Fields] AND "virus"[All Fields]) AND "infections"[All Fields])) OR "chikungunya virus infections"[All Fields])) OR (((("chikungunya fever"[MeSH Terms] OR ("chikungunya"[All Fields] AND "fever"[All Fields])) OR "chikungunya fever"[All Fields]) OR (("infection"[All Fields] AND "chikungunya"[All Fields]) AND "virus"[All Fields])) OR "infection chikungunya virus"[All Fields])) OR (((("chikungunya fever"[MeSH Terms] OR ("chikungunya"[All Fields] AND "fever"[All Fields])) OR "chikungunya fever"[All Fields]) OR ("chickungunya"[All Fields] AND "fever"[All Fields])) OR "chickungunya fever"[All Fields])) OR ((("chikungunya fever"[MeSH Terms] OR ("chikungunya"[All Fields] AND "fever"[All Fields])) OR "chikungunya fever"[All Fields]) OR ("chickungunya"[All Fields] AND "fevers"[All Fields]))) OR (((("chikungunya fever"[MeSH Terms] OR ("chikungunya"[All Fields] AND "fever"[All Fields])) OR "chikungunya fever"[All Fields]) OR ("fever"[All Fields] AND "chickungunya"[All Fields])) OR "fever chickungunya"[All Fields])) AND ((((("inflammation"[MeSH Terms] OR ((("inflammation"[MeSH Terms] OR "inflammation"[All Fields]) OR "inflammations"[All Fields]) OR "inflammation s"[All Fields])) OR ((("inflammation"[MeSH Terms] OR "inflammation"[All Fields]) OR (("innate"[All Fields] AND "inflammatory"[All Fields]) AND "response"[All Fields])) OR "innate inflammatory response"[All Fields])) OR ((("inflammation"[MeSH Terms] OR "inflammation"[All Fields]) OR (("inflammatory"[All Fields] AND "response"[All Fields]) AND "innate"[All Fields])) OR "inflammatory response innate"[All Fields])) OR ((("inflammation"[MeSH Terms] OR "inflammation"[All Fields]) OR (("innate"[All Fields] AND "inflammatory"[All Fields]) AND "responses"[All Fields])) OR "innate inflammatory responses"[All Fields])) OR "inflammatory arthropathy"[All Fields])) AND ((((((((((((((((((((((((((((((((((((((("arthritis, rheumatoid"[MeSH Terms] OR ((("arthritis, rheumatoid"[MeSH Terms] OR ("arthritis"[All Fields] AND "rheumatoid"[All Fields])) OR "rheumatoid arthritis"[All Fields]) OR ("rheumatoid"[All Fields] AND "arthritis"[All Fields]))) OR "spondylitis, ankylosing"[MeSH Terms]) OR (((("spondylitis, ankylosing"[MeSH Terms] OR ("spondylitis"[All Fields] AND "ankylosing"[All Fields])) OR "ankylosing spondylitis"[All Fields]) OR ("spondyloarthritis"[All Fields] AND "ankylopoietica"[All Fields])) OR "spondyloarthritis ankylopoietica"[All Fields])) OR (((("spondylitis, ankylosing"[MeSH Terms] OR ("spondylitis"[All Fields] AND "ankylosing"[All Fields])) OR "ankylosing spondylitis"[All Fields]) OR ("ankylosing"[All Fields] AND "spondylarthritis"[All Fields])) OR "ankylosing spondylarthritis"[All Fields])) OR (((("spondylitis, ankylosing"[MeSH Terms] OR ("spondylitis"[All Fields] AND "ankylosing"[All Fields])) OR "ankylosing spondylitis"[All Fields]) OR ("ankylosing"[All Fields] AND "spondylarthritides"[All Fields])) OR "ankylosing spondylarthritides"[All Fields])) OR ((("spondylitis, ankylosing"[MeSH Terms] OR ("spondylitis"[All Fields] AND "ankylosing"[All Fields])) OR "ankylosing spondylitis"[All Fields]) OR ("spondylarthritides"[All Fields] AND "ankylosing"[All Fields]))) OR (((("spondylitis, ankylosing"[MeSH Terms] OR ("spondylitis"[All Fields] AND "ankylosing"[All Fields])) OR "ankylosing spondylitis"[All Fields]) OR ("spondylarthritis"[All Fields] AND "ankylosing"[All Fields])) OR "spondylarthritis ankylosing"[All Fields])) OR ((("spondylitis, ankylosing"[MeSH Terms] OR ("spondylitis"[All Fields] AND "ankylosing"[All Fields])) OR "ankylosing spondylitis"[All Fields]) OR ("ankylosing"[All Fields] AND "spondylitis"[All Fields]))) OR (((("spondylitis, ankylosing"[MeSH Terms] OR ("spondylitis"[All Fields] AND "ankylosing"[All Fields])) OR "ankylosing spondylitis"[All Fields]) OR ("spondylarthritis"[All Fields] AND "ankylopoietica"[All Fields])) OR "spondylarthritis ankylopoietica"[All Fields])) OR (((("spondylitis, ankylosing"[MeSH Terms] OR ("spondylitis"[All Fields] AND "ankylosing"[All Fields])) OR "ankylosing spondylitis"[All Fields]) OR ("bechterew"[All Fields] AND "disease"[All Fields])) OR "bechterew disease"[All Fields])) OR (((("spondylitis, ankylosing"[MeSH Terms] OR ("spondylitis"[All Fields] AND "ankylosing"[All Fields])) OR "ankylosing spondylitis"[All Fields]) OR ("bechterew s"[All Fields] AND "disease"[All Fields])) OR "bechterew s disease"[All Fields])) OR (((("spondylitis, ankylosing"[MeSH Terms] OR ("spondylitis"[All Fields] AND "ankylosing"[All Fields])) OR "ankylosing spondylitis"[All Fields]) OR ("bechterews"[All Fields] AND "disease"[All Fields])) OR "bechterews disease"[All Fields])) OR (((("spondylitis, ankylosing"[MeSH Terms] OR ("spondylitis"[All Fields] AND "ankylosing"[All Fields])) OR "ankylosing spondylitis"[All Fields]) OR (("marie"[All Fields] AND "struempell"[All Fields]) AND "disease"[All Fields])) OR "marie struempell disease"[All Fields])) OR (((("spondylitis, ankylosing"[MeSH Terms] OR ("spondylitis"[All Fields] AND "ankylosing"[All Fields])) OR "ankylosing spondylitis"[All Fields]) OR (("marie"[All Fields] AND "struempell"[All Fields]) AND "disease"[All Fields])) OR "marie struempell disease"[All Fields])) OR (((("spondylitis, ankylosing"[MeSH Terms] OR ("spondylitis"[All Fields] AND "ankylosing"[All Fields])) OR "ankylosing spondylitis"[All Fields]) OR ("rheumatoid"[All Fields] AND "spondylitis"[All Fields])) OR "rheumatoid spondylitis"[All Fields])) OR (((("spondylitis, ankylosing"[MeSH Terms] OR ("spondylitis"[All Fields] AND "ankylosing"[All Fields])) OR "ankylosing spondylitis"[All Fields]) OR ("spondylitis"[All Fields] AND "rheumatoid"[All Fields])) OR "spondylitis rheumatoid"[All Fields])) OR (((("spondylitis, ankylosing"[MeSH Terms] OR ("spondylitis"[All Fields] AND "ankylosing"[All Fields])) OR "ankylosing spondylitis"[All Fields]) OR ("spondylitis"[All Fields] AND "ankylopoietica"[All Fields])) OR "spondylitis ankylopoietica"[All Fields])) OR (((("spondylitis, ankylosing"[MeSH Terms] OR ("spondylitis"[All Fields] AND "ankylosing"[All Fields])) OR "ankylosing spondylitis"[All Fields]) OR ("ankylosing"[All Fields] AND "spondyloarthritis"[All Fields])) OR "ankylosing spondyloarthritis"[All Fields])) OR ((("spondylitis, ankylosing"[MeSH Terms] OR ("spondylitis"[All Fields] AND "ankylosing"[All Fields])) OR "ankylosing spondylitis"[All Fields]) OR ("ankylosing"[All Fields] AND "spondyloarthritides"[All Fields]))) OR ((("spondylitis, ankylosing"[MeSH Terms] OR ("spondylitis"[All Fields] AND "ankylosing"[All Fields])) OR "ankylosing spondylitis"[All Fields]) OR ("spondyloarthritides"[All Fields] AND "ankylosing"[All Fields]))) OR (((("spondylitis, ankylosing"[MeSH Terms] OR ("spondylitis"[All Fields] AND "ankylosing"[All Fields])) OR "ankylosing spondylitis"[All Fields]) OR ("spondyloarthritis"[All Fields] AND "ankylosing"[All Fields])) OR "spondyloarthritis ankylosing"[All Fields])) OR "spondylarthritis"[MeSH Terms]) OR (("spondylarthritis"[MeSH Terms] OR "spondylarthritis"[All Fields]) OR "spondylarthritides"[All Fields])) OR ((("spondylarthritis"[MeSH Terms] OR "spondylarthritis"[All Fields]) OR ("spinal"[All Fields] AND "arthritis"[All Fields])) OR "spinal arthritis"[All Fields])) OR (("spondylarthritis"[MeSH Terms] OR "spondylarthritis"[All Fields]) OR ("spinal"[All Fields] AND "arthritides"[All Fields]))) OR ((("spondylarthritis"[MeSH Terms] OR "spondylarthritis"[All Fields]) OR ("arthritis"[All Fields] AND "spinal"[All Fields])) OR "arthritis spinal"[All Fields])) OR "arthritis, psoriatic"[MeSH Terms]) OR (((("arthritis, psoriatic"[MeSH Terms] OR ("arthritis"[All Fields] AND "psoriatic"[All Fields])) OR "psoriatic arthritis"[All Fields]) OR ("psoriasis"[All Fields] AND "arthritic"[All Fields])) OR "psoriasis arthritic"[All Fields])) OR (((("arthritis, psoriatic"[MeSH Terms] OR ("arthritis"[All Fields] AND "psoriatic"[All Fields])) OR "psoriatic arthritis"[All Fields]) OR ("arthritic"[All Fields] AND "psoriasis"[All Fields])) OR "arthritic psoriasis"[All Fields])) OR ((("arthritis, psoriatic"[MeSH Terms] OR ("arthritis"[All Fields] AND "psoriatic"[All Fields])) OR "psoriatic arthritis"[All Fields]) OR ("psoriatic"[All Fields] AND "arthritis"[All Fields]))) OR (((("arthritis, psoriatic"[MeSH Terms] OR ("arthritis"[All Fields] AND "psoriatic"[All Fields])) OR "psoriatic arthritis"[All Fields]) OR ("psoriasis"[All Fields] AND "arthropathica"[All Fields])) OR "psoriasis arthropathica"[All Fields])) OR (((("arthritis, psoriatic"[MeSH Terms] OR ("arthritis"[All Fields] AND "psoriatic"[All Fields])) OR "psoriatic arthritis"[All Fields]) OR ("psoriatic"[All Fields] AND "arthropathy"[All Fields])) OR "psoriatic arthropathy"[All Fields])) OR (((("arthritis, psoriatic"[MeSH Terms] OR ("arthritis"[All Fields] AND "psoriatic"[All Fields])) OR "psoriatic arthritis"[All Fields]) OR ("arthropathies"[All Fields] AND "psoriatic"[All Fields])) OR "arthropathies psoriatic"[All Fields])) OR (((("arthritis, psoriatic"[MeSH Terms] OR ("arthritis"[All Fields] AND "psoriatic"[All Fields])) OR "psoriatic arthritis"[All Fields]) OR ("arthropathy"[All Fields] AND "psoriatic"[All Fields])) OR "arthropathy psoriatic"[All Fields])) OR (((("arthritis, psoriatic"[MeSH Terms] OR ("arthritis"[All Fields] AND "psoriatic"[All Fields])) OR "psoriatic arthritis"[All Fields]) OR ("psoriatic"[All Fields] AND "arthropathies"[All Fields])) OR "psoriatic arthropathies"[All Fields])) OR "arthritis"[MeSH Terms]) OR ((("arthritis"[MeSH Terms] OR "arthritis"[All Fields]) OR "arthritides"[All Fields]) OR "polyarthritides"[All Fields])) OR (("arthritis"[MeSH Terms] OR "arthritis"[All Fields]) OR "polyarthritis"[All Fields])) OR ((("arthritis"[MeSH Terms] OR "arthritis"[All Fields]) OR "arthritides"[All Fields]) OR "polyarthritides"[All Fields]))*

**Embase**

*('Chikungunya virus' OR 'Chikungunya fever' OR 'Chikungunya virus infection' OR 'Chikungunya' OR 'Fever, Chikungunya' OR 'Chikungunya virus infections') AND ('inflammation' OR 'inflammatory response' OR 'innate inflammatory response' OR 'inflammatory arthropathy') AND ('rheumatoid arthritis' OR 'ankylosing spondylitis' OR 'spondyloarthritis' OR 'psoriatic arthritis' OR 'Bechterew disease' OR 'Marie-Strümpell disease' OR 'spinal arthritis' OR 'spinal arthritides')*

**LILACS**

*("Vírus Chikungunya" OR "Febre Chikungunya" OR "Infecção por Vírus Chikungunya" OR "Chikungunya" OR "Fever Chikungunya" OR "Infecciones por Virus Chikungunya" OR "Chikungunya Fever" OR "Fiebre Chikungunya") AND ("Inflamação" OR "Resposta Inflamatória Inata" OR "Artrite Inflamatória" OR "Artrite" OR "Artrite Reumatoide" OR "Resposta Inata Inflamatória") AND ("Artrite Reumatoide" OR "Espondilite Anquilosante" OR "Artrite Psoriática" OR "Espondiloartrite Anquilosante" OR "Espondilite Anquilosante" OR "Artrite Espinhal" OR "Artrite Espinal" OR "Spondylarthritis" OR "Artrite Espinal" OR "Doença de Bechterew" OR "Doença de Marie-Strümpell")*

**Cochrane Library**

*ID	Search	Hits*

*#1	MeSH descriptor: [Chikungunya virus] explode all trees*

*#2	MeSH descriptor: [Chikungunya Fever] explode all trees*

*#3	MeSH descriptor: [Inflammation] explode all trees*

*#4	MeSH descriptor: [Arthritis, Rheumatoid] explode all trees*

*#5	MeSH descriptor: [Spondylitis, Ankylosing] explode all trees*

*#6	MeSH descriptor: [Arthritis, Psoriatic] explode all trees*

*#7	MeSH descriptor: [Spondylarthritis] explode all trees*

*#8	#1 OR #2*

*#9	#4 OR #5 OR #6 OR #7*

*#10	#8 AND #3 AND #9*
