# Supplementary material for: Patients with chikungunya meeting criteria for inflammatory rheumatic diseases: a systematic literature review and meta-analysis
Source: Adv Rheumatol. 2025 Sep 24;65:38. doi: 10.1186/s42358-025-00471-6 (PMC13488800; doi:10.1186/s42358-025-00471-6)
Supplement: Supplementary file 3 [file 42358_2025_471_MOESM4_ESM.docx]

***Supplementary Material 4 – Excluded studies on full text lecture with respective reason for exclusion of search strategy***

| ***Title*** | ***Reference*** | ***Reason for exclusion*** |
| --- | --- | --- |
| A report of 21 cases of rheumatoid arthritis following Chikungunya fever. A mean follow-up of two years | *Bouquillard et al, 2009* | ***Serie cases*** |
| Chronic inflammatory arthritis with persisting bony erosions in patients following chikungunya infection | *Chaaithanya et al, 2014* | ***Serie cases*** |
| Inflammatory biomarkers, disease activity index, and self-reported disability may be predictors of chronic arthritis after chikungunya infection: brief report | *Sepúlveda-Delgado et al, 2016* | ***Serie cases*** |
| Persistent Arthralgia Associated with Chikungunya Virus Outbreak, US Virgin Islands | *Feldstein et al, 2017* | ***Wrong patient population*** |
| Seroprevalence of Anti-Chikungunya IgG Antibodies among Rheumatoid Arthritis (RA) Patients | *Verma et al, 2019* | ***Wrong setting*** |
| Investigating the seroprevalence of Chikungunya virus antibodies in new rheumatoid arthritis patients at tworheumatology clinics in Kingston, Jamaica | *Bailey et al, 2018* | ***Wrong setting*** |
| Acute and Chronic Clinical Features of Chikungunya virus infection in patients with rheumatic disorders | *Caballero-Uribe et al, 2016* | ***Wrong setting*** |
| Musculoskeletal manifestations of chikungunya fever in elderly patients - ChikBrasil Cohort | *Dantas et al, 2018* | ***Wrong patient population*** |
| Chikungunya outbreak in Brazil: Demographic and Clinical characterization of 723 patientes - ChikBrasil Cohort | *Duarte et al,2017* | ***Wrong patient population*** |
| Chikungunya Fever Outbreak in Brazil: Preliminary  Assessment in a Cohort of Patients with Rheumatological  Manifestations | *Marques et al, 2016* | ***Wrong patient population*** |
| Identification of Factors Associated With Chronic Chikungunya  Disease in Patients in Grenada, West Indies | *Heath et al, 2016* | ***Wrong patient population*** |
| Anti‐chikungunya IgG antibody positivity and development of rheumatoid arthritis:  results from the Malaysian epidemiological investigation of rheumatoid arthritis (MyEIRA) study | *Tan et al, no date* | ***Wrong patient population*** |
| Post-chikugunya chronic arthritis - Sharing of Bangladesh experience over one year follow up of 60 patients | *Hossain et al, 2020* | ***Wrong setting*** |
| Measures of disease severity predict disability and quality of life differently in rheumatoid arthritis and chronic chikungunya disease | *Watson et al, 2020* | ***Wrong setting*** |
