# Supplementary material for: Patients with chikungunya meeting criteria for inflammatory rheumatic diseases: a systematic literature review and meta-analysis
Source: Adv Rheumatol. 2025 Sep 24;65:38. doi: 10.1186/s42358-025-00471-6 (PMC13488800; doi:10.1186/s42358-025-00471-6)
Supplement: Supplementary file 4 [file 42358_2025_471_MOESM5_ESM.docx]

**Supplementary material 5 - Meta-analysis values**

|  |  | **Chikungunya** | | |
| --- | --- | --- | --- | --- |
|  | **Studies combined** | **Population** | **Sample** | **Proportion (95% CI)** |
| **Inflammatory arthropathy** | 38 | 12524 | 4324 | 0.5807 [0.4845; 0.6769] |
| Q | 54764.47 |  |  |  |
| p | <0.01 |  | | |
| I2 | 99.9% |  |  |  |
| Tau2 | 0.0899 |  |  |  |
| **Inflammatory arthropathy (clinical + laboratory subgroup)** | 14 |  |  | 0.5887 [0.4159; 0.7615] |
| Q | 33425.74 |  |  |  |
| I2 | 100.0% |  |  |  |
| Tau2 | 0.1080 |  |  |  |
| **Inflammatory arthropathy (clinical subgroup)** | 5 |  |  | 0.6340 [0.4012; 0.8668] |
| Q | 636.63 |  |  |  |
| I2 | 99.4% |  |  |  |
| Tau2 | 0.0655 |  |  |  |
| **Inflammatory arthropathy (laboratory subgroup)** | 19 |  |  | 0.5615 [0.4251; 0.6979] |
| Q | 9993.70 |  |  |  |
| I2 | 99.8% |  |  |  |
| Tau2 | 0.0907 |  |  |  |
| **Rheumatoid arthritis** | 10 | 2099 | 240 | 0.1418 [0.0593; 0.2244] |
| Q | 223.18 |  |  |  |
| p | < 0.0001 |  |  |  |
| I2 | 96.0% |  |  |  |
| Tau2 | 0.0168 |  |  |  |
| **Rheumatoid arthritis (ACR/1987 subgroup)** | 4 |  |  | 0.1024 [0.0000; 0.2480] |
| Q | 52.02 |  |  |  |
| I2 | 94.2% |  |  |  |
| Tau2 | 0.0215 |  |  |  |
| **Rheumatoid arthritis (ACR/EULAR 2010 subgroup)** | 6 |  |  | 0.1698 [0.0678; 0.2719] |
| Q | 100.63 |  |  |  |
| I2 | 95.0% |  |  |  |
| Tau2 | 0.0150 |  |  |  |
| **Ankylosing spondylitis** | 3 | 1052 | 36 | 0.0523 [0.0000; 0.1227] |
| Q | 32.84 |  |  |  |
| p | < 0.0001 |  |  |  |
| I2 | 93.9% |  |  |  |
| Tau2 | 0.0032 |  |  |  |
| **Ankylosing spondylitis (New York criteria for AS)** | 2 |  |  | 0.0860 [0.0000; 0.1802] |
| Q | 3.57 |  |  |  |
| I2 | 72% |  |  |  |
| Tau2 | 0.0036 |  |  |  |
| **Spondyloarthritis** | 5 | 711 | 86 | 0.1340 [0.0419; 0.2262] |
| Q | 86.92 |  |  |  |
| p | < 0.0001 |  |  |  |
| I2 | 95.4% |  |  |  |
| Tau2 | 0.0100 |  |  |  |
| **Psoriatic arthritis** | 4 | 1220 | 25 | 0.0623 [0.0000; 0.1734] |
| Q | 17.70 |  |  |  |
| p | 0.0005 |  |  |  |
| I2 | 83.1% |  |  |  |
| Tau2 | 0.0120 |  |  |  |
| **Psoriatic arthritis (CASPAR subgroup)** | 2 |  |  | 0.1348 [0.0000; 0.3968] |
| Q | 16.78 |  |  |  |
| I2 | 94.0% |  |  |  |
| Tau2 | 0.0337 |  |  |  |
| **Psoriatic arthritis (ACR subgroup)** | 2 |  |  | 0.0101 [0.0021; 0.0180] |
| Q | 0.91 |  |  |  |
| I2 | 0.0% |  |  |  |
| Tau2 | 0 |  |  |  |
