# Supplementary material for: Patients with chikungunya meeting criteria for inflammatory rheumatic diseases: a systematic literature review and meta-analysis
Source: Adv Rheumatol. 2025 Sep 24;65:38. doi: 10.1186/s42358-025-00471-6 (PMC13488800; doi:10.1186/s42358-025-00471-6)
Supplement: Supplementary file 5 [file 42358_2025_471_MOESM6_ESM.docx]

**Supplementary material 6**

Sensitivity analysis
